# Nephrotic syndrome: A twist in the tail

**DOI:** 10.4103/0971-4065.45296

**Published:** 2008-10

**Authors:** Vinay Sakhuja, Vivekanand Jha, Amanjit Bal

**Affiliations:** 1 **Chair:** Dean and Professor of Nephrology, PGIMER; 2 **Clinical Discussant:** Additional Professor of Nephrology, PGIMER; 3 **Pathology Discussant:** Assistant Professor of Histopathology, PGIMER

## The Case

A 55 year old housewife was admitted with progressive anasarca. The history went back about 3-4 months when she started experiencing increasing body swelling along with a progressive fall in urine output. The swelling had started from legs, and progressed to involve rest of the body including the face. Associated symptoms included generalized weakness and exertional breathlessness.

There was no urinary symptom including dysuria or hematuria. Orthopnea or PND were absent, as were fever; abdominal pain or diarrhea; headache, nausea or vomiting, visual or auditory symptoms; rashes, photosensitivity, or hair loss.

She did not have diabetes, hypertension or other major past illness including jaundice. A history of pain and swelling in both knee joints of several years’ duration had been recorded for which he used to consult local doctors. A history of “dry mouth” with oral ulcers has been noted by one observer. She had 6 children and had no addictions.

Examination revealed a fully conscious, normally oriented person with massive anasarca; pulse was 80 beats/min (low volume), and distal pulses were not palpable (attributed to extensive edema). Carotids, femoral and brachial pulses could be felt. Blood pressure was 100/60 mm Hg and respiratory rate 20/min. No pallor, cyanosis, or lymphadenopathy were noted and jugular veins were not engorged. Abdominal examination revealed ascites; lungs showed reduced breath sounds at both bases; heart sounds were muffled and neurologic examination did not reveal any abnormality. Vaginal exam showed atrophic uterus and full fornices due to ascites.

**Table:** Investigations

	31/07/2007	06/08/2007	08/08/2007
Hb.	11.2	12.2	10.8
TLC	9000	9200	15200
DLC	66/27/5/2	80/17/2/1	85/12/2/1
ESR			56mm/h
PBF	N/N		N/N
	P/A		P/A
Plat	317 × 10^3^		192 × 10^3^

**Table:** 

	31/07/2007	01/08/2007	08/08/2007
Na^+^	129	136	136
K^+^	4.0	3.7	4.6
Cl	90	95	97
Urea	65	59.76	54
Creat	1.1	0.98	0.94
Ca^+2^		7.19	7.43
Phosphate		4.6	3.8
Alk Ph		196	172
AST/ALT		25/18	37/28
Bil	0.3	0.25	0.14
Protein		2.8	3.12
Alb.		0.98	0.9
RBS	80		
LDL-C		188	

Urine: Alb ++/tr; Sugar nil; M/E occ pus cells; 10-12

RBCs (catheterized sample). culture sterile

24hr urine protein: 1.3 gm and 960 mg

Pl. fluid: P: 200 mg/dl, S: 96 mg/dl; TLC-60/cmm, DLC-distorted morphology, sterile

Ascitic fluid: P 60 mg/dl; TLC - 30/cmm, DLC-80/20; sterile

ECG: low voltage complexes; CXR: b/l pleural effusion

USG Abdomen: RK 9.9 cm; LK 10.2 cm; B/L moderate pl. effusion; and ascites.

Compression USG: no e/o DVT in both LL. Extreme subcutaneous edema noted.

2D Echo: LVED 3.2 cm, LVPW 1.5 cm, IVS 1.75 cm; Ejection Fraction 80%; RVED 1.2 cm; Ao root 2.2 cm; mitral valve E/A 0.5; no RWMA, no effusion/clot/vegetation

**Fig. 1:**
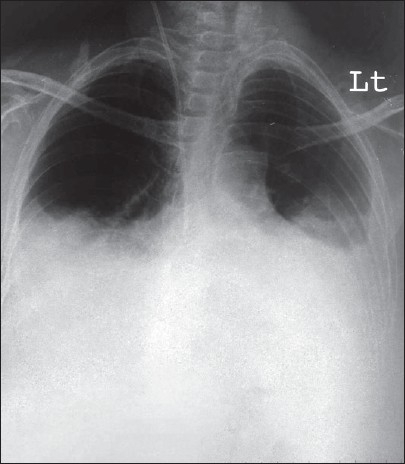
Chest skiagram showing bilateral pleural effusion

Chest skiagram: bilateral pleural effusion [Fig. 1]

HIV, HBsAg, anti-HCV: non-reactive

Urine BJP: -ve; Serum/urine electrophoresis: no “M” band.

PT: 14” (C-13); PTI - 93%; APTT - 31” (C.25” - 32”)

C3: 193.00 (90-180) mg/dl; C4: 32.90 (15- 57) mg/dl

ANA (IF): Negative

pH 7.32; PaO_2_ 68; PaCO_2_ 25; HCO_3_ 14

A central line was inserted, pleural and ascitic taps were done. She was given parenteral diuretics without much success. Albumin infusion was considered but finances were a problem. She developed sudden onset tachypnea, hypotension and tachycardia on 09/08/2007 night, went rapidly downhill and died in a matter of 3 hours despite resuscitative efforts.

**Unit Diagnosis:** Nephrotic syndrome, pulmonary thromboembolism

## Clinical Discussion

**Dr. Vivekanand Jha:** This data base shows a middle aged female who presented with progressive edema, anasarca, serous effusions, she had severe hypovolemia without tachycardia and proteinuria; investigations showed moderately elevated cholesterol and some elevation of alkaline phosphatase. Echocardiogram has shown left ventricular hypertrophy and diastolic dysfunction. She suddenly developed dyspnoea and hypotension and died rapidly.

Her basic symptom was severe edema. The differential diagnosis of edema forming states is limited to heart failure, chronic liver, kidney disease and malnutrition. There is no evidence of congestive heart failure or chronic liver disease such as cirrhosis that could have caused portal hypertension, liver failure and edema. Apart from severe hypoalbuminemia in this otherwise healthy adult female there was no evidence of any other nutritional deficiencies. She did not have any other protein losing state including protein losing enteropathy due to diarrhoea. That leaves us with kidney disease. The evidence that supports presence of kidney disease is the presence of proteinuria causing. A variety of the diseases of the kidney can give rise to edema. This patient had proteinuria and hypercholesterolemia in the presence of a normal blood pressure, bland urine sediments and a normal glomerular filtration rate, the classical description of nephrotic syndrome. The finding of a relatively low value of proteinuria (about 1 gm/d) can be explained by the presence of severe hypoalbuminemia that restricts the amount of protein that can leak into urine. The presence of any nephrotic syndrome almost immediately takes us to a suspicion of a glomerular disease. The main glomerular diseases that lead to a nephrotic syndrome are listed in Table 1. This patient had no clinical clue to pont towards a secondary glomerular disease on the face of it. In the primary glomerular disease the first three are the ones that present predominantly with a pure nephrotic syndrome. The latter are proliferative glomerular diseases which can present as nephrotic syndrome but they also have some nephritic features (hematuria, reduced GFR) and hence are unlikely [Table 2].

**Table 1:** Causes of nephrotic syndrome

Primary glomerular disease	Secondary glomerular diseases
Minimal change disease	Diabetic nephropathy
Membranous nephropathy	Infection-associated
Focal segmental glomerulosclerosis	Malignancy associated
Mesangiocapillary GN	Multisystem disease
Post-infectious GN	Drugs, allergens, venoms
IgA nephropathy	Heredofamilial

**Table 2:** Clinical presentation of primary glomerular diseases

	Nephrotic Features	Nephritic Features
MCD	++++	-
Membranous GN	++++	+
FSGS	+++	++
Fibrillary GN	+++	++
MesProl GN	++	++
MPGN	++	+++
DPGN	+	++++
Crescentic GN	+	++++

So it would mean that this patient could have either a minimal change disease or membranous nephropathy. Membranous nephropathy is far more common than minimal change disease in adults, so if it is a primary glomerular disease, it is likely to be one of these two. Of the secondary causes, some can be straightaway ruled out; these include medications, allergen, immunization and infections. Of the systemic diseases, lupus nephritis can be ruled out because of absence of any clinical features or supportive laboratory findings. The last one is amyloidosis and we will come back to it. The next set of causes is malignancies; membranous nephropathy is known to be associated with the variety of malignancies especially in the elderly. However, the clinical and investigative profile does not favor this possibility.

**Fig. 2:**
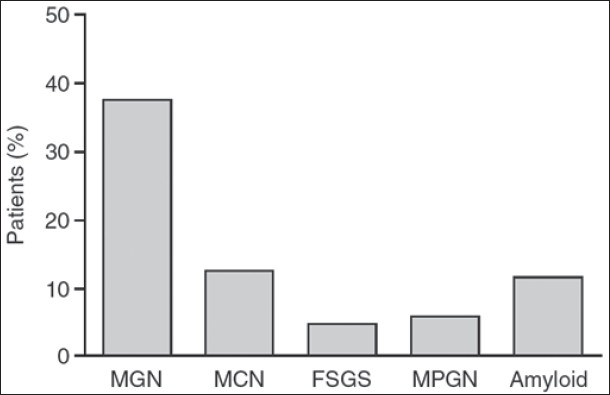
Distribution of causes of nephrotic syndrome in the elderly (Cameron, 1996)

If one looks at the causes of nephrotic syndrome in the elderly, we find this paper published 12 years ago by Stewart Cameron.^1^ He looked at over 1000 patients of nephrotic syndrome over the age of 60; the most frequent cause was MN, followed by minimal change nephropathy (13%). The third commonest cause was amyloid, only slightly less frequent (12.5%) than minimal change nephropathy [Fig. 2]. Amyloidosis is a condition in which there is excessive extracellular deposition of fibrils in various organs. It can be caused by about 20 different proteins, with the two most frequent being AA and AL types. AA amyloid occurs in chronic inflammatory diseases such as rheumatoid arthritis or in India, tuberculosis. There was some history of joint pains but not enough to qualify for the diagnosis of inflammatory joint disease. The AL or so called primary amyloid is much more frequent in individuals over the age of 50. Males outnumber female by 2:1. The symptoms are exactly the ones she had, weight loss, fatigue, shortness of breath, and edema. Kidneys are the most commonly affected organ and presentation is with nephrotic syndrome and bland urinary sediment; hypertension is much less common, and hypercholesteremia is less severe as in this case despite the severe hypoalbuminemia. Amyloid involves a number of other organs. There is mild elevation of alkaline phosphatase which could suggest involvement of the liver with an infiltrative process. The chief cardiac findings in this patient were low voltage electrocardiogram, a concentric left ventricular hypertrophy indicating increased LV mass (in the absence of hypertension) and a diastolic dysfunction as indicated by the mitral inflow assessment showing A>E. The main echo finding was the increased LV mass. The commonest cause for this is hypertension which was not present in this case. Another cause could be hypertrophic cardiomyopathy which was ruled out by echocardiography. Next is list of infiltrative disorders such as glycogen storage disorders, sarcoidosis, hemochromatosis, and Fabry's disease, but the most prominent of these is amyloidosis. She had no evidence of any of the other infiltrative disorders. A number of studies have shown that the combination of increased left ventricular mass and a reduced voltage on ECG has a very high sensitivity and the specificity approaching almost 100% for cardiac amyloid.^2^–^4^ Diastolic dysfunction present in this case is another hallmark of cardiac amyloid. Upto 88% show restrictive pattern on Doppler mitral inflow assessment and it increases with disease severity.^5^ The classical description of sparkling pattern being the typical echocardiographic appearance for amyloid has been found to be a very insensitive indicator; the sensitivity is only 25-35%.^6^,^7^,^9^ Moreover, this spectral pattern was described only with older echocardiographic machines which were relatively crude. They did not apply tissue harmonics and so made the myocardium appear coarser than usual. The newer machines that apply tissue harmonics do not show this pattern, hence its absence is not surprising. The final organ system that is possibly involved is the autonomic nervous system, shown by the absence of tachycardia in the face of reduced blood pressure.

The next area of curiosity is the cause of death. The most likely possibility is that of is thromboemobolic complications. They develop as a result of complex metabolic abnormalities which alter the balance towards prothrombotic state; both arterial and venous arteries can be involved. The risk increases exponentially once the serum albumin is less than 2 g/dl, and these patients need to be put on anticoagulation during hospitalization, something that was not done in this case. Arterial circulation is involved more frequently in children than in adults. Patients with membranous nephropathy, lupus nephritis and amyloid are particularly vulnerable to develop venous thrombotic disorders. Immobilization as occurs in a hospitalised individual further increases this risk. The prevalence in different series has been noted to be as high as 45%. So it is quite likely that this patient had silent thromboses and threw an embolus in the pulmonary circulation causing sudden onset dyspnea, followed quickly by hypotension and death in a matter of hours. The extent of thrombosis is hard to guess, one would certainly expect to see an embolus in the pulmonary artery, but it could be much more extensive.

**Dr. Jha's diagnosis:**

- Systemic Amyloidosis (possibly AL) with nephrotic syndrome, cardiac, and hepatic involvement and autonomic neuropathy
- Thromboembolic complications secondary to nephrotic syndrome
- Cause of death: Pulmonary embolism

**Dr. Sakhuja:** Dr.Jha is made out a very convincing case for amyloidosis possibly primary as the most likely diagnosis with pulmonary thrombo-embolism. This patient did not receive any anticoagulant. When such patients are admitted to the hospital with severe hypoalbuminemia we generally put them on prophylactic anticoagulation when they are bedridden for short periods of time; possibly this was not done because a biopsy was being planned However, I think in those circumstances I think it should have been done.

**Dr. KL Gupta, Professor of Nephrology:** I would lean towards the diagnosis of occult malignancy, most likely non-Hodgkin extra nodular lymphoma with membranous nephropathy.

**Dr. A Rajvanshi, Professor of Cytology:** This patient had a history of pain and swelling both knees for several years which was not investigated. It could be rheumatoid arthritis that might have led to the amyloidosis. The fine needle aspiration of the abdominal fat would have helped to make the diagnosis of amyloidosis.

**Dr. Sakhuja:** Yes that is an alternative possibility that can be considered. Dr. Jha discounted the possibility of Sjogren syndrome because of the lack of a proper history but even that could explain some joint pains, the dryness of the mouth, oral ulceration and that is also known to be associated with a nephrotic syndrome although that is an uncommon combination. If you get a nephrotic syndrome it is usually a membranous nephropathy but most frequently Sjogren’s will present with chronic interstitial renal damage which presents with renal tubular function defects like distal renal tubular acidosis for which this patient did not have any evidence.

**Dr. KS Chugh, Emeritus Professor of Nephrology:** Rheumatoid arthritis gives rise to amyloid disease when it is generally an advanced burnt out disease and is quite obvious. Just history of joint pains does not mean a rheumatoid arthritis. Even in a case of amyloid disease joint pains are well known and therefore some kind of pains in the joints may be part and parcel of primary amyloid disease.

## Autopsy Findings

**Dr. Amanjit Bal:** A partial autopsy was performed: Each pleural cavity yielded 1000ml and the peritoneal cavity yielded 2000ml of straw colored fluid. Pericardial cavity was within normal limits.

**Fig. 3:**
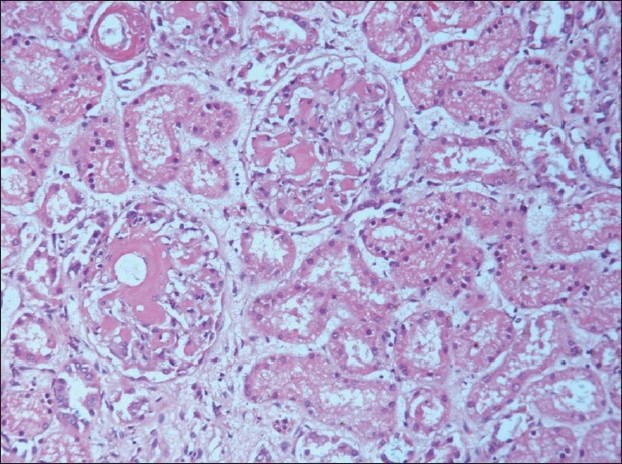
Photomicrograph showing extensive deposits of pale pink eosinophilic material in the extracellular space in the glomeruli. (H&E, ×150)

**Fig. 4:**
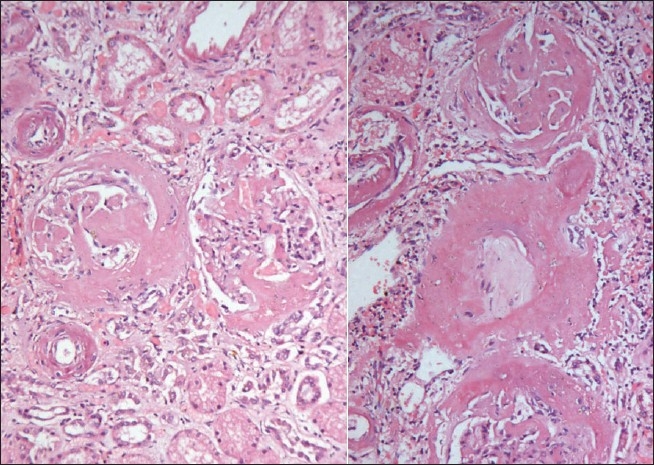
Amyloid deposits in the interstitium and blood vessels (H&E, ×150 left, ×440 right)

**Fig. 5:**
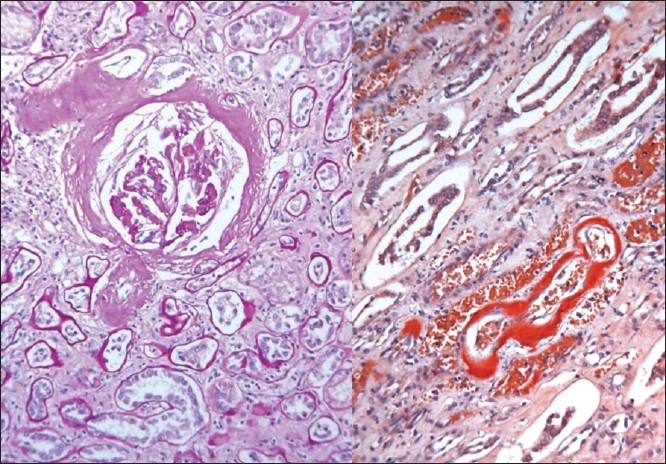
The amyloid shows light PAS positivity (left) and bright congophilia (right)

**Fig. 6:**
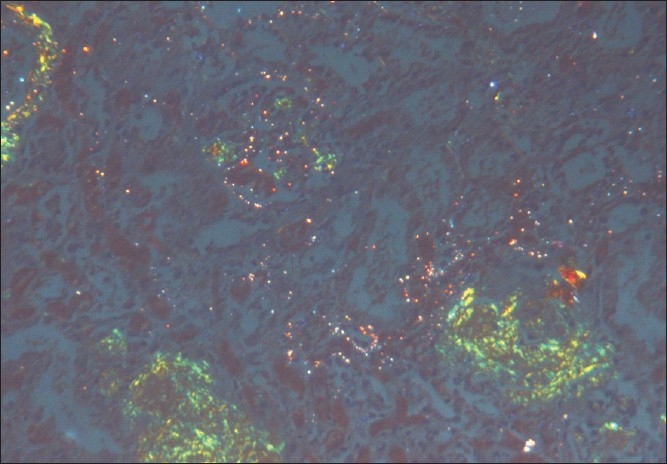
Apple green birefringence seen under polarized light

**Fig. 7:**
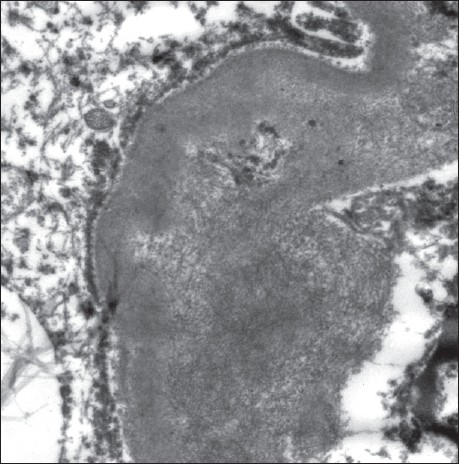
Electron microscopy show randomly arranged extracellular amyloid fibrils

The kidneys weighed 250gm and were of normal size. The external appearance was blotchy and granular with a simple cortical cyst 0.5cm of diameter. On cutting, the edges were sharp with well defined margins; there was medullary congestion. On microscopy, glomeruli showed diffuse and nodular deposition of pale amorphous eosinophilic material in the mesangium, basement membrane and along the Bowman's capsule. Similar deposits were seen in afferent arterioles, and small and medium sized blood vessels [Figs. 3 and 4]. This material was weakly PAS positive, and brightly congophilic [Fig. 5]. Exposure to polarized light revealed apple green birefringence [Fig. 6]. Tubules showed atrophy, hyaline casts, and features of acute tubular necrosis. Interstitium shows patchy inflammatory infiltrate. Electron microscopy showed randomly arranged thin, non-branching fibrils with dots within mesangium, basement membrane and arterioles [Fig. 7].

**Fig. 8:**
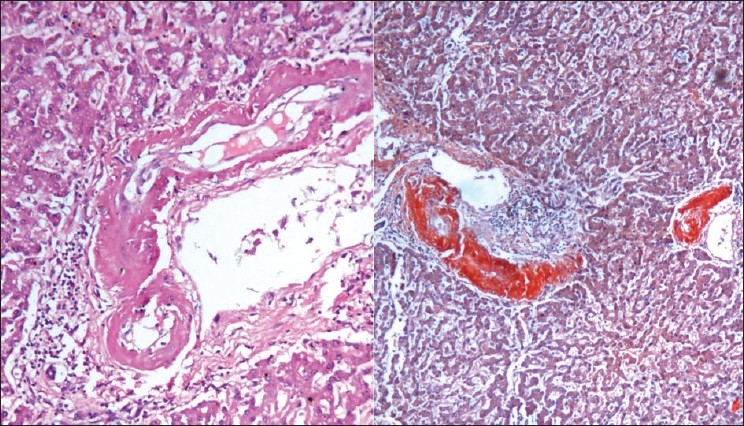
Amyloid deposits in the hepatic blood vessels and around sinusoids, H&E ×150 (left) and Congo red stain ×150 (right)

The liver weighed 1050gm, and on cutting showed exaggerated mottling. Microscopy showed sinusoidal dilatation and deposition of amyloid in vessels. Spleen was firm with sharp cut margins, microscopy showed extensive amyloid deposit in red and white pulps [Fig. 8].

**Fig. 9:**
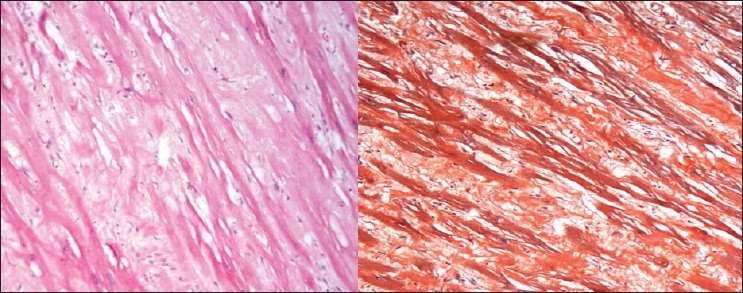
Extracellular amyloid deposits around cardiac myofibrils. H&E ×150 (left) and Congo red stain ×150 (right)

The heart was enlarged with shiny visceral pericardium. Both atria were dilated with shiny endocardium. There was biventricular wall thickening. The right and left ventricular free walls were 0.7 cm 1.7 cm thick respectively. Microscopy showed extensive interstitial and perivascular deposition of amyloid, at places surrounding each myocyte leading to atrophy of myocytes [Fig. 9]. The valvular endocardium also shows nodular deposits of amyloid.

**Fig. 10:**
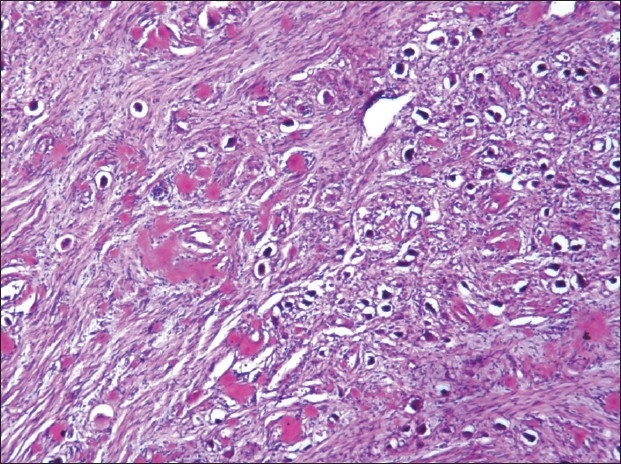
Photomicrograph shows amyloid deposits in spinal ganglia around neuronal cell bodies. (PAS ×150)

The spinal ganglia and peripheral nerves showed extensive deposition of amyloid and evidence of loss of myelin [Fig. 10].

Pancreas was firm and atrophic; and microscopy shows extensive amyloid deposits. Islets of Langerhans were preserved. Blood vessels of oesophagus, stomach, Intestine, adrenals, uterus and ovaries showed amyloid deposits. On immunohistochemistry, the amyloid protein was negative for AA and for Kappa and Lambda light chains. The bone marrow showed a normal representation of haematopoetic elements; there was no excess of plasma cells or amyloid deposits.

**Fig. 11:**
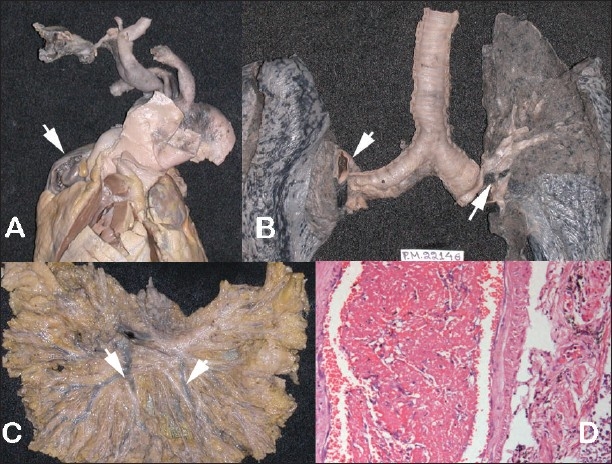
A-C Gross photograph showing extensive thrombosis (arrows) in the A. cardiac auricle, B. Pulmonary arteries and C. Mesenteric veins and D. Shows the photomicrograph of one such thrombus.

There was widespread venous thrombosis involving the superior vena cava, inferior vena cava, hepatic veins, superior mesenteric vein, splenic vein, and right renal vein. Both pulmonary arteries contained emboli [Fig. 11].

## Characterization of Amyloid

**Dr. Purnananda Guptasarma, Scientist, Institute of Microbial Technology, Chandigarh**

**Fig. 12:**
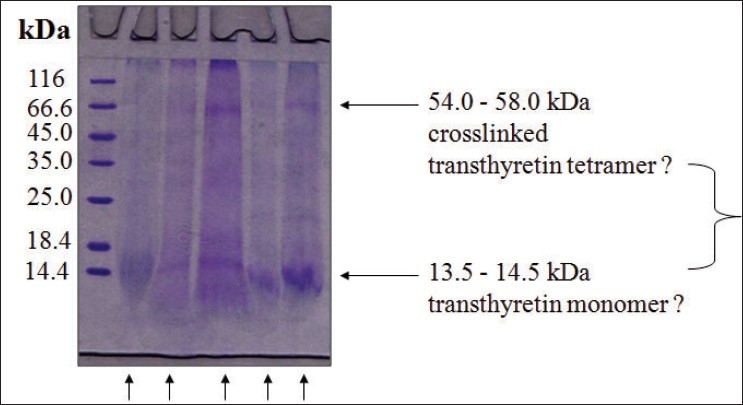
SDS-PAGE of amyloid extracted from the cardiac tissue using different conditions (arrows) shows the transthyretin bands of expected sizes

**Fig. 13:**
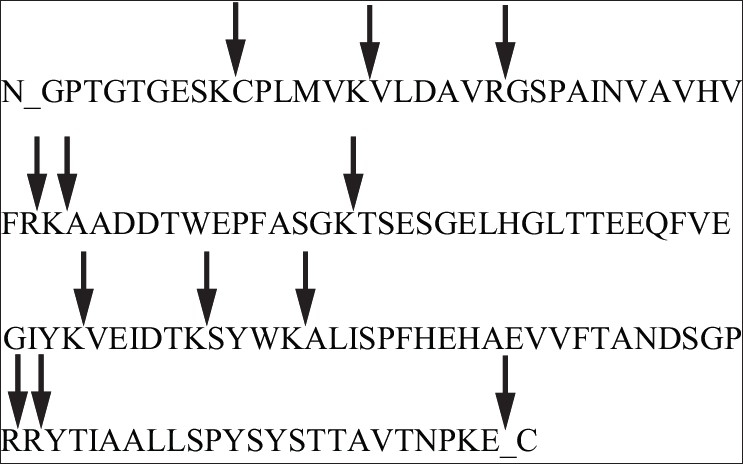
Amino acid sequence of transthyretin with trypsin cleavage sites (arrows)

**Fig. 14:**
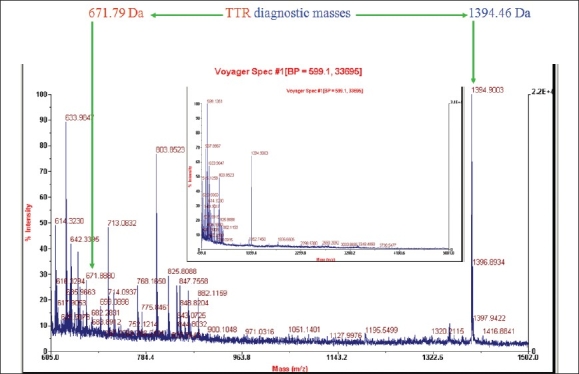
Mass spectroscopy on the tryptic digest of the band extracted from the gel

**Fig. 15:**
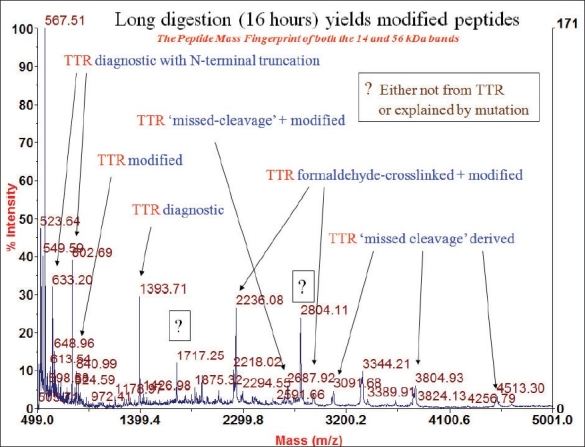
Mass spectroscopy on the prolonged (16 hour) tryptic digest of the band extracted from the gel shows additional peaks, suggesting the possibility of modified (mutated) peptide fragments

We extracted the amyloid from the cardiac tissue using the protocol of Layfield^8^. Fig. 12 shows a representative sodium dodecyl sulphate polyacrylamide gel electrophoresis (SDS-PAGE) using different conditions. They all produced essentially identical results, and the PAGE shows is a protein somewhere between 14.4 to 18.4 KD. Another protein of about 54 to 58 kDa can be seen [Fig. 12]. This is consistent with transthyretin (TTR) protein which has a molecular weight of about 13763 Da and so these bands could represent TTR monomer and a cross linked TTR tetramer. We extracted the protein from the gel using in-gel tryptic digestion, digested it with proteases and did mast spectrometry and peptide mass fingerprinting. The results from both the bands were identical suggesting that they were both the same proteins only differentially cross linked. Formaldehyde modifies these proteins making them appear slightly bigger. Unlike most proteins which loose their structure when they deposit as amyloids proteins, the transthyretin protein is known to retain most of its native structure in the amyloid state. In the amyloid, the tetramers associate through helix-swapping among subunits non- covalently bringing the tetramers together. So we have the situation of the cross linked tetramers associated non-covalently. Trypsin, which cleaves proteins just after lysine and arginine residues can be predicted to yield 13 fragments, whose molecular masses can be predicted on the basis of the known sequence of TTR [Fig. 13]. The two masses that you see here are amongst those. One can't expect to see all the tryptic fragments because of the crosslinking and modifications. So if one sees a few and not all of these peaks, and knowing that you expected only one protein from the tissue one can be sure of its identity. Essentially the same mass fingerprint was seen from the both bands and led to the identification of TTR because these two diagnostic masses of 676.79 and 1394.46 Da dominate the spectrum. When the spectrum is blown up to show 500-5000 Da range, the only band is the 1394 Da band which only comes from transthyretin [Fig. 14]. So we established that this protein that forms the amyloid is transthyretin. On long (16 hour) digestion, we found some couple of masses that can't be explained and could be mis-cleavages due to TTR mutations, or crosslinking produced due to formaldehyde [Fig. 15]. These would change the expected mass of a peptide and the observed mass would expect depend on the nature of the mutation or linkage.

## Final Diagnosis

- Transthyretin associated amyloidosis (Familial amyloid polyneuropathy) involving heart, kidneys, spinal ganglia and peripheral nerves, spleen, pancreas, liver and vascular involvement of other organs.
- Thromboembolic complications involving: superior vena cava, bilateral pulmonary vessels, inferior vena cava, hepatic veins, splenic vein, superior mesenteric vein and right renal vein.

Transthyretin associated amyloidosis includes two differential diagnosis, senile cardiac amyloidosis and familial amyloid polyneuropathy. Senile amyloidosis is seen in those over the age of 80 and is due to deposition of normal or wild type of transthyretin. Heart is the predominant organ involved. In kidneys, renal cortex is never involved and hence nephrotic syndrome does not develop. Familial amyloid polyneuropathy develops due to deposition of mutated transthyretin, and the predominant involvement is seen in nerves and heart. Renal and splenic involvement is uncommon. Over 100 mutations have been described. So the clinical phenotype in this case fits with that of familial amyloid polyneuropathy. Initially this condition was considered to be peculiar to certain endemic regions but now has been reported from non-endemic areas from around the world. It is inherited in an autosomal dominant fashion. The age of onset is younger in the endemic areas and ranges from 20-40 years. In non-endemic areas it is seen in individuals in their 50s to late 60s. After onset of clinical symptoms, the disease is almost universally fatal over the next decade. Patients have been diagnosed as familial amyloid polyneuropathy even in the absence of family history; this has been explained because of the variable genetic penetrance.

**Dr. Sakhuja:** So we did end up with amyloidosis but of a different kind from what we thought. As far as the neuropathy is concerned none of that was also picked up during life; we required a more detailed neurological examination. I have a query for Dr. Guptasarma. You mentioned the difficulties that you had with identifying the protein in case of formalin preserved tissues. In which form would you prefer them to be to make identification easier?

**Dr. Guptasarma:** Actually in this case it helped that formalin prevents other proteins from being extracted because then you essentially extract the amyloid. If you don't have formalin, you would about 40-50,000 proteins. Formalin cross linking can be reversed using a certain set of conditions where you use very high salt and very high temperature and extensive incubation. With amyloid, it should be possible to extract the amyloid and reverse the cross linking to some extent.

**Dr. Sanjay Jain, Professor of Internal Medicine:** It is very hard for me to believe that patient has presentation of a very advanced nephrotic syndrome but no symptomatology of polyneuropathy.

**Dr. Jha:** I would like to thank Dr. Guptasarma for helping us identify the protein. Amyloid formation is known to be associated with at least 20 different proteins but the ones that predominantly involve the heart are only two: AL and transthyretin. Transthyretin is seen in specific clinical situations which are either Senile Amyloidosis and Familial Amyloid Polyneuropathy.

**Dr. Chugh:** Chambers described in the 50s and 60s a large series of familial polyneuropathy due to amyloid and in those cases they did not have really the features of nephrotic syndrome. I don't know if more cases have been described but this is one of the biggest series which can be quoted.

**Dr. Sakhuja:** The lesson that we learnt today is that when you have excluded secondary amyloid, not all of what is left is primary amyloid. I would also like to emphasize that liver transplantation is the definitive treatment for TTR amyloid if it can be diagnosed early.
